# Oxysterol-binding protein-like 2 contributes to the developmental progression of preadipocytes by binding to β-catenin

**DOI:** 10.1038/s41420-021-00503-2

**Published:** 2021-05-17

**Authors:** Tianming Wang, Tianyu Zhang, Youzhi Tang, Hongshun Wang, Qinjun Wei, Yajie Lu, Jun Yao, Yuan Qu, Xin Cao

**Affiliations:** 1 Department of Medical Genetics, School of Basic Medical Science, Nanjing Medical University, Nanjing, China; 2 Jiangsu Cancer Hospital, Nanjing Medical University, Nanjing, China; 3 Jiangsu Key Laboratory of Xenotransplantation, Nanjing Medical University, Nanjing, China

**Keywords:** Development, Obesity

## Abstract

Oxysterol-binding protein-like 2 (OSBPL2), also known as oxysterol-binding protein-related protein (ORP) 2, is a member of lipid transfer protein well-known for its role in regulating cholesterol homeostasis. A recent study reported that OSBPL2/ORP2 localizes to lipid droplets (LDs) and is associated with energy metabolism and obesity. However, the function of OSBPL2/ORP2 in adipocyte differentiation is poorly understood. Here, we report that OSBPL2/ORP2 contributes to the developmental progression of preadipocytes. We found that OSBPL2/ORP2 binds to β-catenin, a key effector in the Wnt signaling pathway that inhibits adipogenesis. This complex plays a role in regulating the protein level of β-catenin only in preadipocytes, not in mature adipocytes. Our data further indicated that OSBPL2/ORP2 mediates the transport of β-catenin into the nucleus and thus regulates target genes related to adipocyte differentiation. Deletion of OSBPL2/ORP2 markedly reduces β-catenin both in the cytoplasm and in the nucleus, promotes preadipocytes maturation, and ultimately leads to obesity-related characteristics. Altogether, we provide novel insight into the function of OSBPL2/ORP2 in the developmental progression of preadipocytes and suggest OSBPL2/ORP2 may be a potential therapeutic target for the treatment of obesity-related diseases.

## Introduction

Oxysterol-binding protein (OSBP) and OSBP-related protein (ORP) or OSBP-like (OSBPL) proteins have been found to contain a conserved OSBP-related domain (ORD) with oxysterol-binding activity and constitute a large family that regulates lipid homeostasis^1^. Several members of the OSBP/ORPs family are linked to obesity-related diseases: ORP8 has previously been reported to play a key role in the development of type II diabetes mellitus (T2DM)^2,3^; ORP11 is suggested to be closely associated with cardiovascular disease risk factors and is expressed at a high level in visceral adipose tissue (VAT) in obese population with metabolic syndrome^4^; and ORP5 controls the synthesis of neutral lipid and thus regulates the lipid droplet (LD) size^5^.

Oxysterol-binding protein-like 2 (OSBPL2/ORP2) is an important ORP that contains an FFAT (diphenylalanine [FF] in an acidic tract [AT]) motif for ER targeting and a functional ORD for lipid binding^6^. Similar to other members of the OSBP/ORPs family, OSBPL2/ORP2 is a lipid transfer protein (LTP)^7^ and has a function in lipid transport and regulation of sterol and phospholipid metabolism^6,8–11^. OSBPL2/ORP2 also regulates the actin cytoskeleton and affects cell adhesion^12^. In addition, OSBPL2/ORP2 is associated with LD formation^13^ and is considered as an important effector in energy metabolism^14^. Recent studies have showed a linkage between OSBPL2/ORP2 and obesity-related disease: the regulation of LD lipolysis required OSBPL2/ORP2^15^, and the *OSBPL2*-disruption resulted in hypercholesterolemia in miniature pig models^16^. However, the role of OSBPL2/ORP2 in the development of obesity-related disease and the underlying regulatory mechanism of adipocyte differentiation have yet to be fully elucidated.

Preadipocyte development is strongly linked to the onset of obesity upon its committed differentiation into an adipocyte. During the developmental process, the morphology of preadipocytes changes, and the function of preadipocytes become similar to that of mature adipocytes, including gaining the capacity for adipogenesis and lipid droplet (LD) storage^17,18^. During this period, pro-adipogenic transcription factors such as PPAR-γ (a preadipocyte marker), together with C/EBPα, commit preadipocytes to differentiate into mature adipose tissue^19^. The Wnt signaling pathway is one of the previously reported regulators of preadipocyte development and adipocyte differentiation. β-Catenin, a component of adherens junctions, plays a key role in cell adhesion^20^ and is an effector in the canonical Wnt signaling pathway. β-Catenin has three distinct domains: the N-terminal fraction, armadillo (ARM) domain, and C-terminal fraction^21,22^. The ARM domain contains 12 armadillo repeats (40 aa) is an important site for the interaction of its partners. The ARM domain also contains the SRP fraction, which is a conserved domain in the SRP1 superfamily that appears to bind to the nuclear envelope and is associated with the nuclear trans-localization^23,24^. It is reported that β-catenin negative regulates adipocyte differentiation by suppressing PPAR-γ and C/EBPα activation, and thus maintains preadipocytes in an undifferentiated state^25^. In the progression of preadipocyte development, β-catenin is phosphorylated upon forming a destruction-bound complex modified with ubiquitin and is ultimately degraded by the proteasome^26^.

Here, we demonstrate that OSBPL2/ORP2 is required for maintaining preadipocytes in the undifferentiated stage. Loss of OSBPL2/ORP2 facilitates 3T3-L1 preadipocyte maturation, and the absence of Osbpl2b leads to the adipose tissue (AT) presenting with much larger LDs and the acquisition of an obesity phenotype by the zebrafish model. Overall, our study provides important insights into the function of OSBPL2/ORP2, and the results strongly suggest that *OSBPL2* might be a novel target for the treatment of obesity-related disease in humans.

## Methods and materials

### Chemicals and antibodies

BODIPY 493/503 (#D3922) and DAPI (#D1306) were purchased from Invitrogen (CA, USA). SKL2001 was purchased from Selleckchem (Shanghai, China). Dexamethasone (#D4902), 3-isobutyl-1-methylxanthine (#I5879), insulin (#I2643), Oil red O (#O9755), MS-222 (#E10521), and Triton X-100 (#T8787) were purchased from Sigma-Aldrich (MO, USA). Antibodies against P42/44 (#9102), β-catenin (#9562), Phospho-β-catenin (#9561), PPAR-γ (#2443), Ubiquitin (#3936), GAPDH (#5174), HA-tag (#3724) as well as secondary HRP-conjugated antibodies against mouse (#7076) and rabbit (#7074) were purchased from CST (MA, USA) and used at 1:1,000 dilution for western blotting. Anti-FLAG-tag antibody (#F1804) and anti-FLAG-tag M2 affinity gel (#A2220) were from Sigma-Aldrich (MO, USA). Antibodies against Plin1 (#A16294), OSBPL2 (#A14199), α-Tubulin (#AC012), β-catenin (#A11512) as well as secondary HRP-conjugated Mouse Anti-Rabbit IgG Light Chain (#AS061) were from ABclonal (Wuhan, China). Antibodies against β-catenin (#BF0319) for zebrafish were from Affinity Biosciences (Changzhou, China). Alexa Fluor 488 donkey anti-mouse IgG and Alexa Fluor 546 donkey anti-rabbit IgG were purchased from Life Technologies (CA, USA).

### Zebrafish maintenance and mutant generation

Zebrafish were grown in buffered reverse osmosis (RO) water with a standard light/dark cycle of 14 h/10 h at 28 °C. Adult zebrafish were fed shrimp twice a day and larvae were fed the same amount of paramecium and egg yolk. Three months post fertilization (mpf), the zebrafish were used to mate and breed. All zebrafish were subjected to standard husbandry procedures and used in accordance with standard procedures and guidelines of the Institutional Animal Care and Use Committee (IACUC) of Nanjing Medical University. The *osbpl2b*-knockout zebrafish were previously generated in our laboratory using the CRISPR/Cas9 gene-editing technique. The guide RNA targeting exon 6 of *osbpl2b* was designed using ZiFiT software (http://zifit.partners.org/ZiFiT/), and sgRNA was co-injected into one-cell stage zebrafish embryos of the Tü wild-type strain, which caused a 5-nucleotide (GAGCT) deletion in exon 6 of *osbpl2b*.

### Cell culture

The 3T3-L1 preadipocytes and HEK293T cells were cultured in Dulbecco’s modified Eagle’s medium (DMEM, Gibco, USA) supplemented with 10% fetal bovine serum (FBS, BI, USA) in a humidified atmosphere containing 5% CO_2_ at 37 °C. For the transient plasmid transfections, the 3T3-L1 preadipocytes were grown on coverslips in 24-well plates overnight. The 3T3-L1 preadipocytes were then transfected with 1 μg plasmid using Lipofectamine 3000 assay (Invitrogen, CA, USA) and incubated for 48 h.

### Generation of stable *Osbpl2*-deficient 3T3-L1 cell lines

*Osbpl2*-knockout 3T3-L1 preadipocytes were generated using the CRISPR/Cas9 gene-editing technique. The guide RNA (CAGCGGCTGGGACTGACTTG) targeting exon 4 of *osbpl2* was designed using CHOPCHOP software (https://chopchop.rc.fas.harvard.edu/), and was packaged into an all-in-one CRISPR lentivirus vector (ABM, Richmond, Canada). WT 3T3-L1 preadipocytes were seeded into 24-well plates at a density of 0.5 × 10^5^ cells/well and after 18 h, they were incubated with lentivirus for 48 h. Monoclonal cells were obtained by selection in puromycin conditioned culture, and sequencing was performed on the genomic DNA, which revealed a 2-nucleotide (AA) deletion in exon 4 of *Osbpl2*. In addition, the effectiveness of *osbpl2*-deficiency was analyzed by western blotting with an anti-OSBPL2 antibody (ABclonal, China).

### Cell treatment

Following a classical method^27^ to induce preadipocytes into mature adipocytes, 3T3-L1 preadipocytes were grown in 6-well plates for 48 h before treatment. The 3T3-L1 preadipocytes were treated with MDI (1 μg/ml 3-isobutyl-1-methylxanthine, 1 μM dexamethasone, and 1 μg/ml insulin) or DI (1 μM dexamethasone and 1 μg/ml insulin) for 48 h, and then the 3T3-L1 cells were maintained in DMEM containing DI and 10% FBS until mature.

### LD staining

The 3T3-L1 preadipocytes were grown on coverslips and followed by treatment according to the experimental plan. After being treated for the appropriate times, the cells were immobilized with 4% paraformaldehyde (PFA) for 15 min and then permeabilized with 0.1% Triton X-100 (Sigma-Aldrich, USA) for 10 min at room temperature. Then, cells were stained with BODIPY 493/503 (used at 1:1000 dilution, Invitrogen, USA) for 10 min to and with DAPI (used at 1:1000 dilution, Invitrogen, USA) for 5 min. Confocal microscopy was used with ZEN software to acquire high-quality images. The larvae were maintained in RO water containing BODIPY 493/503 (used at 1:500 dilution, Invitrogen, USA) for 30 min and then anesthetized with MS-222 (Sigma-Aldrich, USA). Images were acquired by fluorescence stereo microscope (Olympus RI2, Japan).

### Oil red O staining

The 3T3-L1 preadipocytes were immobilized with 4% PFA for 15 min, and then washed with 60% isopropyl alcohol for 5 min. Then the cells were soaked in 0.3% oil red O solution (Sigma-Aldrich, USA) for 15 min and washed with 60% isopropyl alcohol for 15 s. After cells were washed with PBS, images were acquired by fluorescence stereo microscope (Olympus RI2, Japan).

### Protein extraction

For the analysis of nucleus fractionation and cytosolic proteins, a nucleus and cytoplasmic extraction assay (#78833, Thermo Scientific, USA) was used according to the protocol. The 3T3-L1 preadipocytes were first washed with cold PBS and harvested with trypsin-EDTA. After cells were centrifugated, the supernatant was discarded to ensure that the pellet was as dry as possible, and then, ice-cold CER I and CER II was added stepwise. The mixture was centrifugated and the supernatant containing the cytosolic fraction was poured into a new tube. Then, the pellets were suspended with ice-cold NER incubated on ice for 40 min with vigorous vortexing for 15 s every 10 min. After centrifugation, the supernatant containing the nucleus proteins was poured into a new tube. Successful separation of the cytosolic and nucleus fractions was routinely validated by western blotting with GAPDH (a cytosolic marker, CST, USA) and HISTONE H3 (a nucleus marker, CST, USA), respectively.

### Western blot analysis

The samples obtained from the cells or tissues lysed with RIPA buffer (Beyotime, China) were separated by 10% SDS-PAGE (Bio-Rad, USA) and transferred onto PVDF membranes (Merck, Germany). The membranes were blocked with TBS-T containing 5% milk powder (skim milk, BD, US) for 2 h and then incubated with primary antibody overnight at 4 °C. The next day, the membranes were incubated with secondary antibodies for 2 h. The bands were visualized with an Odyssey® CLx Imaging System (LI-COR) or by a chemiluminescence method.

### Total RNA isolation and real-time fluorescence quantitative PCR analysis

Total RNA was extracted by TRIzol reagent (Invitrogen, USA), and cDNA was synthesized using a HiScript II one step RT-PCR kit (Vazyme, China). The quantitative real-time PCR (qRT-PCR) was performed on a StepOne Plus system (Applied Biosystems, USA) with ChamQ SYBR qPCR Master Mix (Vazyme, China). The primers used to amplify the specific genes are listed in Supplementary Table S1.

### Coimmunoprecipitation (Co-IP)

HEK293T cells were co-transfected with HA-tagged *β-catenin* plasmid and FLAG-tagged *OSBPL2* or truncated proteins plasmid for 48 h. Then samples were washed three times with ice-cold PBS and lysed for 30 min with RIPA lysis buffer (Beyotime, China) containing 0.2 mM PMSF. The lysate was centrifuged for 30 min at 13,500 rpm and 4 °C. The supernatant was collected and incubated overnight with anti-FLAG-tag M2 affinity gel (Sigma-Aldrich, USA) at 4 °C. The lysate from the cells transfected with HA-tagged *β-catenin* was extracted and immunoprecipitated using anti-FLAG-tag M2 affinity gel (Sigma-Aldrich, USA) as a negative control. The beads were washed more than five times, added to 6×SDS loading buffer, and boiled at 100 °C for 10 min. Then, the samples were assayed by western blotting. For the proteomics experiments, the lysate was extracted from the HEK293T cells transfected with FLAG-tagged *OSBPL2* and was immunoprecipitated with anti-FLAG-tag M2 antibody or immunoprecipitated with normal IgG as a negative control. The protein bands were retrieved and subjected to LC-MS/MS analysis (Applied Protein Technology Co., Ltd, Shanghai, China).

### Immunofluorescence staining

The 3T3-L1 preadipocytes were grown on coverslips and followed by a treatment according to the experimental plan. After being treated for the appropriate times, the cells were immobilized with 4% PFA for 15 min, permeabilized with 0.1% Triton X-100 (Sigma-Aldrich, USA) for 10 min at room temperature, and then blocked with 10% goat serum (Jackson Immuno-Research, USA) for 1 h at room temperature. The coverslips were incubated overnight with primary antibody at 4 °C: rabbit anti-HA (used at 1:800 dilution, CST), and mouse anti-FLAG (used at 1:200 dilution, Sigma-Aldrich). Secondary antibodies were incubated for 1 h at 37 °C: Alexa Fluor 488 donkey anti-mouse IgG (used at 1:1000 dilution, Life Technologies), and Alexa Fluor 546 donkey anti-rabbit IgG (used at 1:1000 dilution, Life Technologies). The cells were incubated with BODIPY 493/503 (used at 1:1000 dilution, Invitrogen) for 10 min at room temperature and with DAPI (used at 1:1000 dilution, Invitrogen) for 5 min. Images were captured by confocal microscopy and visualized with ZEN software.

### Immunohistochemistry

Zebrafish brain and liver paraffin sections obtained from zebrafish at 4 mpf were subjected to gradient ethanol dewaxing and followed by antigen repair. Then, the samples were blocked with 10% goat serum (#005-000-121, Jackson Immuno-Research, USA) for 1 h at room temperature, and incubated overnight with primary antibodies at 4 °C: anti-β-catenin (used at 1:100 dilution, Affinity Biosciences) and anti-OSBPL2 (used at 1:100 dilution, ABclonal). The next day, the samples were incubated with secondary antibodies for 1 h at 37 °C: HRP-conjugated goat anti-rabbit IgG (used at 1:200 dilution, CST). Images were acquired by microscopy.

### Computational 3D structural modeling

Biological macromolecular structures of OSBPL2 (ID: 5ZM8) and β-catenin (ID: 1QZ7) were obtained from the RCSB PDB website (https://www.rcsb.org/). Protein-protein docking was performed using GRAMM-X software (http://vakser.compbio.ku.edu/resources/gramm/grammx/) and the macromolecular structures were visualized with PyMOL software (https://pymol.org/2/).

### Statistical analysis

The number and size of LDs were analyzed with Image J software. All data are presented as the mean ± SD. Student’s *t*-test was used for comparisons between two independent sample groups, one-way analysis of variance (ANOVA) was used for single-factor comparisons among multiple groups, and two-way ANOVA was used for two-factor comparisons among multiple groups; *P* < 0.05 was regarded as significant.

## Results

### *Osbpl2* deletion increased both the size and number of LDs in 3T3-L1 preadipocytes

OSBPL2/ORP2 is a member of the LTP family that participates in lipid transport and plays a role in maintaining intracellular cholesterol homeostasis. To explore the effect of OSBPL2/ORP2 on obesity-related disease, we generated *Osbpl2-*deficient 3T3-L1 preadipocytes by using the CRISPR–Cas9 gene-editing technique, through which amino acid sequences were truncated and altered (Supplementary Fig. S1a, b).

**Fig. 1:**
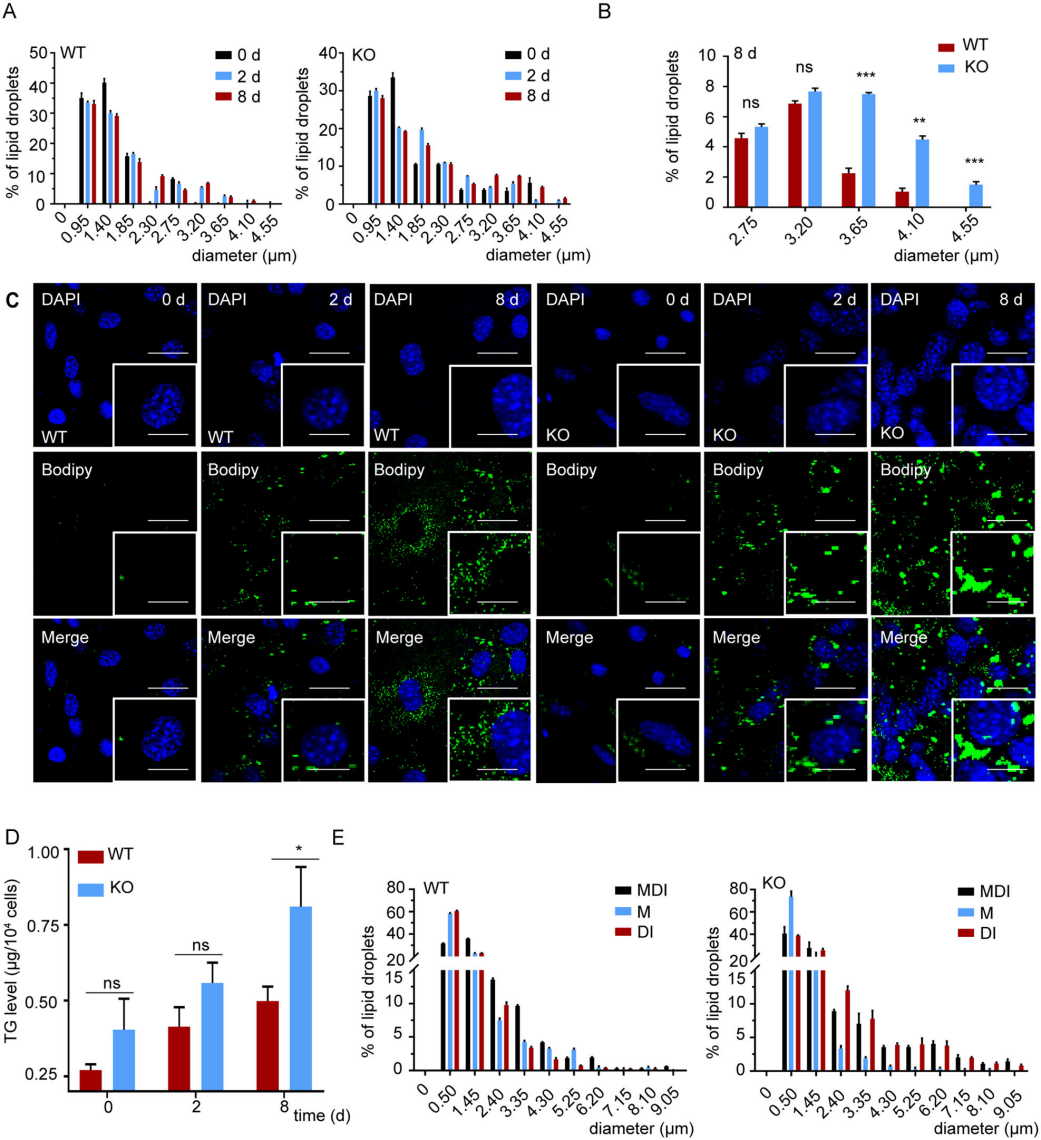
OSBPL2/ORP2 is associated with the development of preadipocytes. The 3T3-L1 preadipocytes were cultured for the indicated periods. **A**–**C** WT cells and KO cells were fixed and stained with BODIPY 493/503 (green) and DAPI (blue) to identify dynamic changes of the LDs. Scale bar, 20 μm (inserts, 10 μm). The distribution of the LDs in the WT and KO cells on day 8 was measured with Image J software. **D** The concentration of TG in the WT cells and KO cells from day 0 to 8 was detected with a TG biochemical detection assay (*n* = 3). **E** The 3T3-L1 preadipocytes were treated with MDI, M, or DI for 48 h and cultured for the indicated periods. MDI: 3-isobutyl-1-methylxanthine (M), dexamethasone (D), and insulin (I). The distribution of the LDs was determined with Image J software to detect the dynamic changes of the LDs in the WT cells and KO cells. The scheme diagram representing the amino acid sequence of OSBPL2 in the KO cells and the WT cells are shown in Supplementary Fig. S1. All data are from three independent experiments. The data are presented as the mean ± SD values (*n* ≥ 3). *P < 0.05; **P < 0.01, ***P < 0.001, ns: not significant.

To observe the dynamic changes in LDs formation, we first cultured WT cells and *Osbpl2*^−/−^ (KO) cells for as long as 8 d without any treatment. A confocal microscope was used, and the LDs were fluorescently stained with BODIPY 493/503 to delineate LD size and distribution patterns as previously described^28^. We confirmed that the size of the LDs increased in the KO cells (Fig. 1A–C). On day 0 and day 2, there were no striking differences between the two different cells, with few LDs observed. However, the LDs fused and became strikingly larger in the KO cells than in the WT cells on day 8 (Fig. 1A–C). Furthermore, we also found a similar trend in which the triglyceride (TG) level increased in the KO cells compared to the WT cells on day 8, as determined using a biochemical TG detection kit (Fig. 1D). These results indicated that OSBPL2/ORP2 may participate in the development of preadipocytes and adipocyte differentiation.

### OSBPL2/ORP2 is required for maintaining 3T3-L1 preadipocytes in the undifferentiated Stage

Preadipocyte development and adipocyte differentiation are delicate and complicated, biological processes. With the treatment of a classical induction cocktail (MDI: 3-isobutyl-1-methylxanthine, dexamethasone, and insulin), 3T3-L1 preadipocytes were induced and to differentiate into mature adipocytes^27,29^. As showed in Supplementary Fig. S1c–e, we found that the level of OSBPL2/ORP2 decreased at an early stage of preadipocyte differentiation, but increased later on, suggesting that OSBPL2/ORP2 could play differential roles at different stages of preadipocyte development and adipocyte differentiation.

To seek further evidence to verify the development of preadipocytes and adipocyte differentiation in WT and KO cells, inducers with different constituents were used. We found that the LDs accumulated to a similar extent on day 8 in the MDI group (Supplementary Fig. S2). One of the components of MDI, 3-isobutyl-1-methylxanthine, can trigger the maturation of preadipocytes into mature adipocytes. We also found no difference on day 8 between the WT cells and KO cells treated only with 3-isobutyl-1-methylxanthine. However, when submaximal stimulation (DI) was applied, the KO cells had greater adipogenic potential than the WT cells (Fig. 1E).

Taken together, our results showed that *Osbpl2* deletion promoted the preadipocyte morphology shift, and indicated that OSBPL2/ORP2 was required for maintaining undifferentiated 3T3-L1 preadipocytes.

### OSBPL2/ORP2 binds to β-catenin and forms a complex

**Fig. 2:**
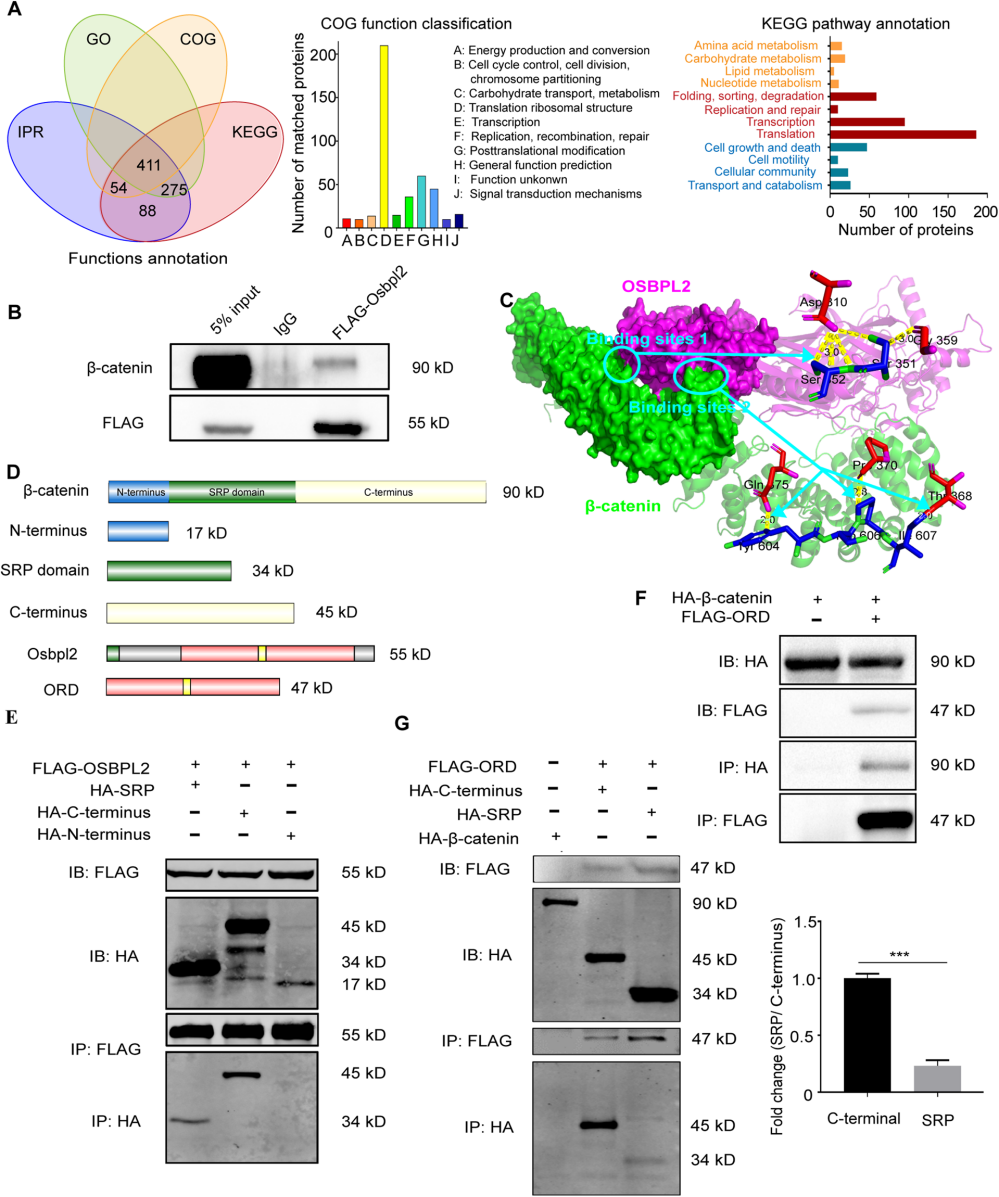
OSBPL2/ORP2 binds to β-catenin. **A** Mass spectrometry data of the HEK293T cells expressing FLAG-tagged OSBPL2 were used to identify and evaluated the OSBPL2 interactome and categorized its components by COG and KEGG analyses. **B** 3T3-L1 preadipocytes were transfected with FLAG-tagged *Osbpl2* plasmid for 48 h. Co-IP assays were used to verify the interaction of FLAG-tagged OSBPL2 with endogenous β-catenin in 3T3-L1 preadipocytes. **C** Panoramic view (right) and amplified view (left) showing the bond between β-catenin (green) and OSBPL2 (rose red). Binding sites 1, ORD in OSBPL2 (Asp-310, Gly-359) and Arm repeats in β-catenin (Ser-351 and Ser-352 residues); binding sites 2, ORD in OSBPL2 (Gln-375, Pro-370, and Thr-368 residues) and Arm repeats in β-catenin (Tyr-604, Pro-606, and Ile-607 residues). **D** Schematic representing the OSBPL2, β-catenin, and truncated proteins. **E** Co-IP assays were used to verify the interaction of OSBPL2 with β-catenin or the truncated (N-terminal, SRP, and C-terminal) fractions in HEK293T cells. HEK293T cells co-expressing the truncated HA-tagged (N-terminal) β-catenin and FLAG-tagged OSBPL2 were used as negative controls. **F** Co-IP assays were used to verify the interaction of β-catenin with the truncated OSBPL2 (ORD). HEK293T cells expressing HA-tagged β-catenin only were used as a negative control. **G** Co-IP assays were used to verify the interaction of the ORD of OSBPL2 with β-catenin or truncated (SRP or C-terminal) fraction. HEK293T cells expressing HA-tagged β-catenin only were used as a negative control. All data are from three independent experiments. The data are presented as the mean ± SD values (*n* ≥ 3). ***P < 0.001.

To explore the underlying mechanism of OSBPL2/ORP2 in preadipocyte development, we identified more than 828 proteins by proteomic analysis of the OSBPL2/ORP2 interactome (Fig. 2A). A COG analysis revealed that the enriched clusters were related to translation, posttranslational modification, and metabolism. A KEGG analysis showed that OSBPL2/ORP2 was associated with translation, transcription, and degradation pathways (Fig. 2A). We found that β-catenin, a component of the Wnt signaling pathway associated with translation, posttranslational modification, and degradation, was among the binding partners of OSBPL2/ORP2. β-Catenin is also known as a suppressor of adipogenesis in the canonical Wnt signaling pathway that activates proliferation-related to genes and inhibits adipogenic genes^30^, which prompted us to test whether β-catenin and OSBPL2/ORP2 are associated with each other at the molecular level. Coimmunoprecipitation (Co-IP) experiments showed specific interaction of OSBPL2 with β-catenin in 3T3-L1 preadipocytes (Fig. 2B and Supplementary Fig. S3a). Next, a 3D model of the two protein structures was constructed, and the binding sites were predicted using PyMOL software. The analysis showed a hydrophobic interaction between the ORD domain in OSBPL2/ORP2 and β-catenin in armadillo/ARM repeat at binding sites 1 and 2 (Fig. 2C).

We next constructed different domains of OSBPL2/ORP2 and β-catenin to verify the binding sites and explore the underlying function in the development of preadipocytes. As shown in Fig. 2D, we constructed the ORD domain of OSBPL2/ORP2, which is known to bind lipids such as sterol. We also truncated β-catenin into three parts: an SRP fraction (aa 142–431), a C-terminal fraction (aa 432–781), and an N-terminal fraction (aa 1–141). The SRP fraction is a conserved domain in the SRP1 superfamily that appears to bind to the nuclear envelope and is associated with trans-localization into the nucleus^24^. As shown in the co-IP results presented in Fig. 2E, OSBPL2/ORP2 binds to β-catenin at its SRP fraction and C terminus. The Co-IP results in Fig. 2F show that the ORD domain of OSBPL2 interacts with β-catenin. We next co-transfected FLAG-tagged ORD with HA-tagged SRP or HA-tagged C terminus. The Co-IP results showed that the ORD in OSBPL2/ORP2 binds to the C terminus but interacted with the SRP fraction slightly (Fig. 2G).

We found that OSBPL2/ORP2 binds to β-catenin and confirmed that this complex mainly depends on the interaction between the ORD domain of OSBPL2/ORP2 and the C terminus (and/or part of the SRP fraction) of β-catenin.

### OSBPL2/ORP2 regulates β-catenin by maintaining the protein level of β-catenin

**Fig. 3:**
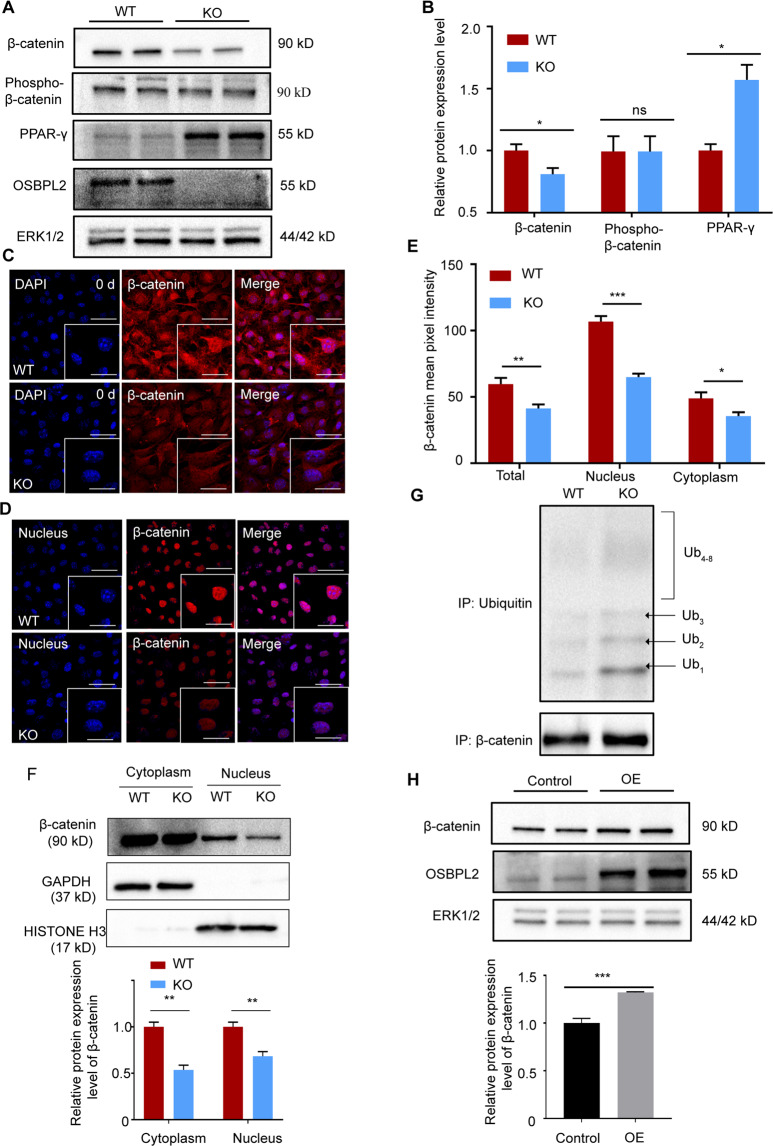
Deletion of *Osbpl2* reduces the β-catenin level in 3T3-L1 preadipocytes. **A**, **B** Lysates of WT and KO cells were immunoblotted for β-catenin, phospho-β-catenin, PPAR-γ, OSBPL2, and ERK1/2 (*n* = 3). The data are normalized to the ERK1/2 control. **C** WT cells and KO cells at day 0 were fixed and immune-stained with anti-β-catenin (red). Scale bar, 50 μm (inserts, 10 μm). **D**, **E** Reduced nuclear localization of β-catenin (imaged by confocal microscope and detected by ZEN software, where a mask is created within the nucleus and the mean pixel intensity of β-catenin within the nucleus is compared to that within the entire cell) related to **C**. Scale bar, 50 μm (inserts, 10 μm). **F** WT cells and KO cells were lysed, and the lysates were separated into cytosol and nucleus fractions. The abundance of β-catenin in the two fractions was assessed by western blotting. HISTONE H3 acted as internal nucleus control, and GAPDH acted as internal cytoplasm control (*n* = 3). **G** β-Catenin were IP with anti-β-catenin antibody in WT preadipocytes or KO preadipocytes and lysates were immunoblotted for ubiquitin detection (*n* = 3). **H** Lysates of 3T3-L1 preadipocytes transfected with empty vector or FLAG-tagged *Osbpl2* plasmid for 48 h were immunoblotted for β-catenin and ERK1/2 detection (*n* = 3). OE = OSBPL2 overexpression. Data are normalized to the ERK1/2 control. All data are from three independent experiments. The data are presented as the mean ± SD values (*n* ≥ 3). *P < 0.05; **P < 0.01, ***P < 0.001, ns: not significant.

Through the degradation pathway, β-catenin is first bound to APC and AXIN to form a complex and is then phosphorylated; finally, it is ubiquitinated and degraded by the proteasome^31,32^. The level of β-catenin was detected to be decreased in the KO cells, while the level of PPAR-γ was increased (Fig. 3A–C). Moreover, this reduction in β-catenin was extensive, not only in the cytoplasm but also in the nucleus (Fig. 3C–F). And we found that β-catenin in the KO cells had more ubiquitin groups than that in the WT cells but had no significant difference in the phospho-β-catenin level (Fig. 3A, B, G). This result indicated that OSBPL2 maintains the protein level of β-catenin by inhibiting its ubiquitylation level. But β-catenin in WT mature adipocytes was at a low level which was similar with KO cells (Supplementary Fig. S3b). We thus hypothesized that expressed OSBPL2/ORP2 can maintain the protein level of β-catenin in preadipocytes but not in mature adipocytes. To verify this finding, FLAG-tagged *Osbpl2* was constructed and transfected into 3T3-L1 preadipocytes. The western blot analysis results showed that overexpressing OSBPL2/ORP2 increased β-catenin at the protein level (Fig. 3H).

Our results showed that OSBPL2/ORP2 bind with β-catenin in a complex, and the absence of OSBPL2/ORP2 led to a decrease in β-catenin, suggesting that OSBPL2/ORP2 is required for maintaining β-catenin.

### OSBPL2 promotes β-catenin translocation into the nucleus

**Fig. 4:**
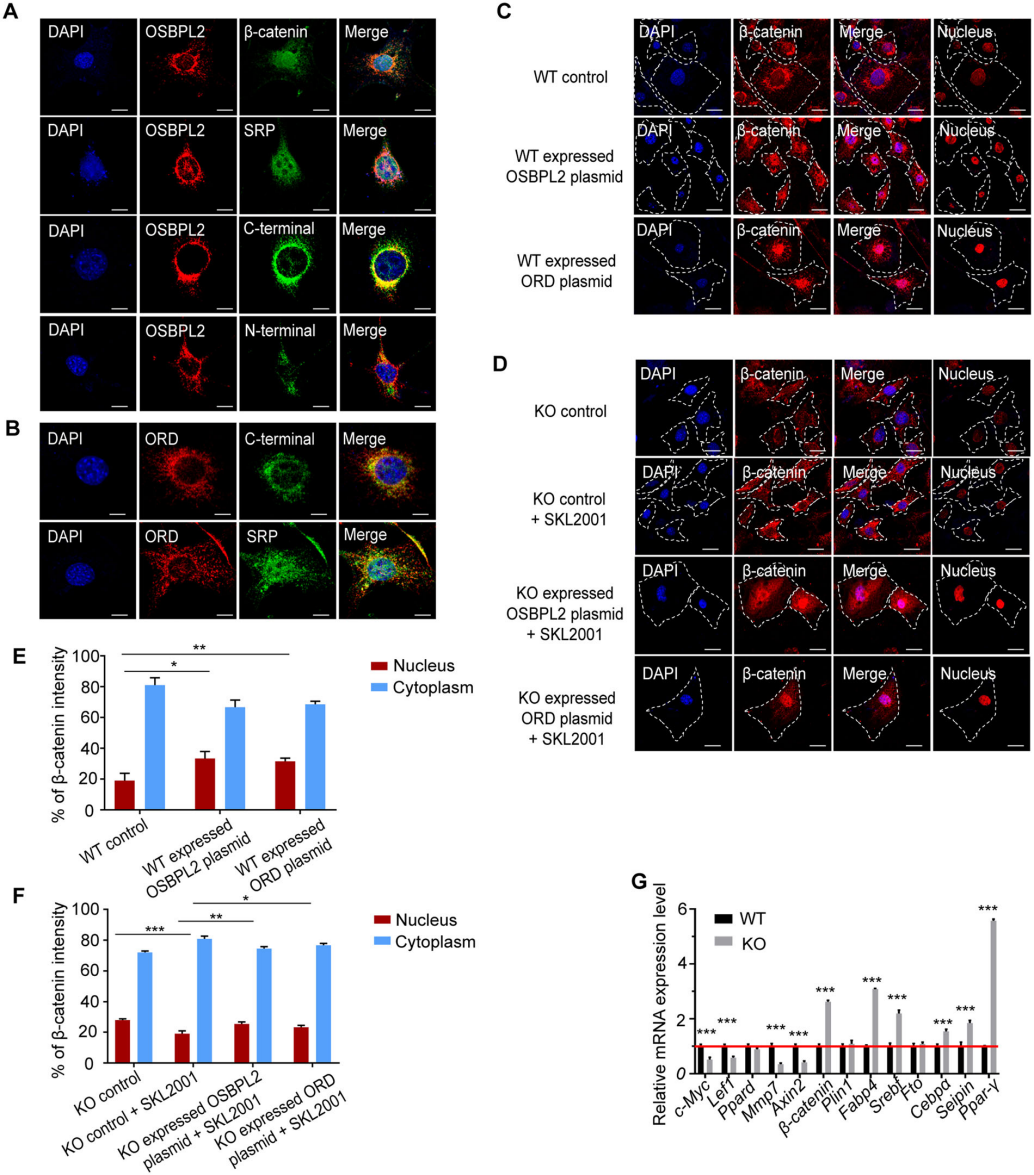
OSBPL2 interacts with β-catenin to facilitate its transport into the nucleus. **A** The 3T3-L1 preadipocytes were co-transfected with 1 μg FLAG-tagged *Osbpl2* plasmid and 1 μg HA-tagged *β-catenin* or 1 μg a truncated *β-catenin* (N-terminal, SRP, or C-terminal) fraction plasmid for 48 h. The cells were fixed and immunostained with anti-HA (green) and anti-FLAG (red). Scale bar, 10 μm. **B** The 3T3-L1 preadipocytes were co-transfected with 1 μg FLAG-ORD plasmid and 1 μg HA-tagged β*-catenin* truncated (SRP and C-terminal) fraction plasmid for 48 h. The cells were fixed and immunostained with anti-HA (green) and anti-FLAG (red). Scale bar, 10 μm. **C**–**F** WT cells and KO cells were transfected with 1 μg empty vector or 1 μg FLAG-tagged *Osbpl2* plasmid and incubated for 48 h. The KO cells were treated with or without 20 μM SKL2001 for 24 h. The cells were fixed and immunostained with anti-β-catenin (red) and DAPI (blue). Scale bar, 50 μm. Nuclear localization of β-catenin was imaged by confocal microscope and detected by ZEN software, where a mask was created within the nucleus and the intensity of β-catenin within the nucleus was compared to that within the entire cell. **G** Real-time RT-PCR was used to analyze the mRNA levels of adipocyte differentiation-related genes in the WT cells and KO cells (*n* = 3). All data are from three independent experiments. The data are presented as the mean ± SD values (*n* ≥ 3). *P < 0.05; **P < 0.01, ***P < 0.001.

OSBPL2 is reported to regulate several nuclear transcription factors^33,34^, and β-catenin is also an important transcriptional activator that regulates the expression of multiple genes downstream^35^. To further explore the function of the OSBPL2-β-catenin complex, FLAG-tagged *Ospbl2* was co-transfected with HA-tagged *β-catenin* or its truncated fractions in 3T3-L1 preadipocytes. The immunofluorescence assay results showed that the ORD in OSBPL2/ORP2 co-localized with the C terminus and part of the SRP fractions in β-catenin as a complex to the nuclear envelope (Fig. 4A, B). These results indicated that the OSBPL2-β-catenin complex is associated with β-catenin trans-localization into the nucleus, which may affect the transcription of downstream genes. We further verified that transfecting *Osbpl2* plasmid or ORD plasmid in WT cells can increase the ratio of intensities of β-catenin within the nucleus versus the entire cell (Fig. 4C, E). Treating with SKL2001^36^ (an β-catenin agonist) in KO cells, the intensity of β-catenin was increased in the cytoplasm and the ratio of the intensity of β-catenin within the nucleus versus the entire cell was decreased compared with that in the KO control cells, but transfecting *Osbpl2* plasmid or ORD plasmid rescued this phenomenon (Fig. 4D, F). In addition, the qRT-PCR results showed that the reduced β-catenin in the nucleus of KO cells caused a change in the transcription level of its downstream genes and thus may affect obesity-related genes via different signaling pathways (Fig. 4G): the β-catenin target genes (such as *c-Myc, Lef1, Ppard, Mmp7,* and *Axin2*) decreased, and some of the obesity-related genes (such as *Fabp4, Srebf, Cebpα, Seipin,* and *Ppar-γ*) increased. We also found that β-catenin mRNA expression level was increased in the KO cells despite lower protein levels, which is supposed to be a compensatory effect. Further study would be performed to explain this point. We also found that treating with SKL2001 in the KO cells partly rescued the phenotypes of cholesterol accumulation and adipose differentiation (Supplementary Fig. S3d–f).

Thus, we concluded that OSBPL2/ORP2 bound to β-catenin into a complex through the binding site in the ORD domain of OSBPL2/ORP2 and the C terminus of β-catenin. The complex restricted β-catenin to the cytoplasm, and β-catenin trans-localized into the nucleus further regulated the downstream adipogenic differentiation gene transcription. *Osbpl2* deletion in preadipocytes reduced β-catenin levels and promoted preadipocyte differentiation into mature adipocyte.

### Reduced β-catenin and increased Ppar-γ in the KO zebrafish

We previously showed that *Osbpl2* deficiency promoted preadipocyte development and adipocyte differentiation via a decrease in β-catenin and upregulation of PPAR-γ in vitro. Next, we selected zebrafish as an animal model to verify whether *Osbpl2* deficiency promotes preadipocyte development and adipocyte differentiation in vivo. Two genes (*osbpl2a* and *osbpl2b*) in zebrafish are homologous with human *OSBPL2*, and *osbpl2b* is an orthologous gene that shares 71.2% homology with human OSBPL2 amino acid sequences^37^. We previously generated *osbpl2b*^−/−^ zebrafish using the CRISPR/Cas9 gene-editing technique, which caused a 5-nucleotide (GAGCT) deletion in exon 6 of *osbpl2b* and resulted in a truncated protein (Supplementary Fig. S4a, b).

**Fig. 5:**
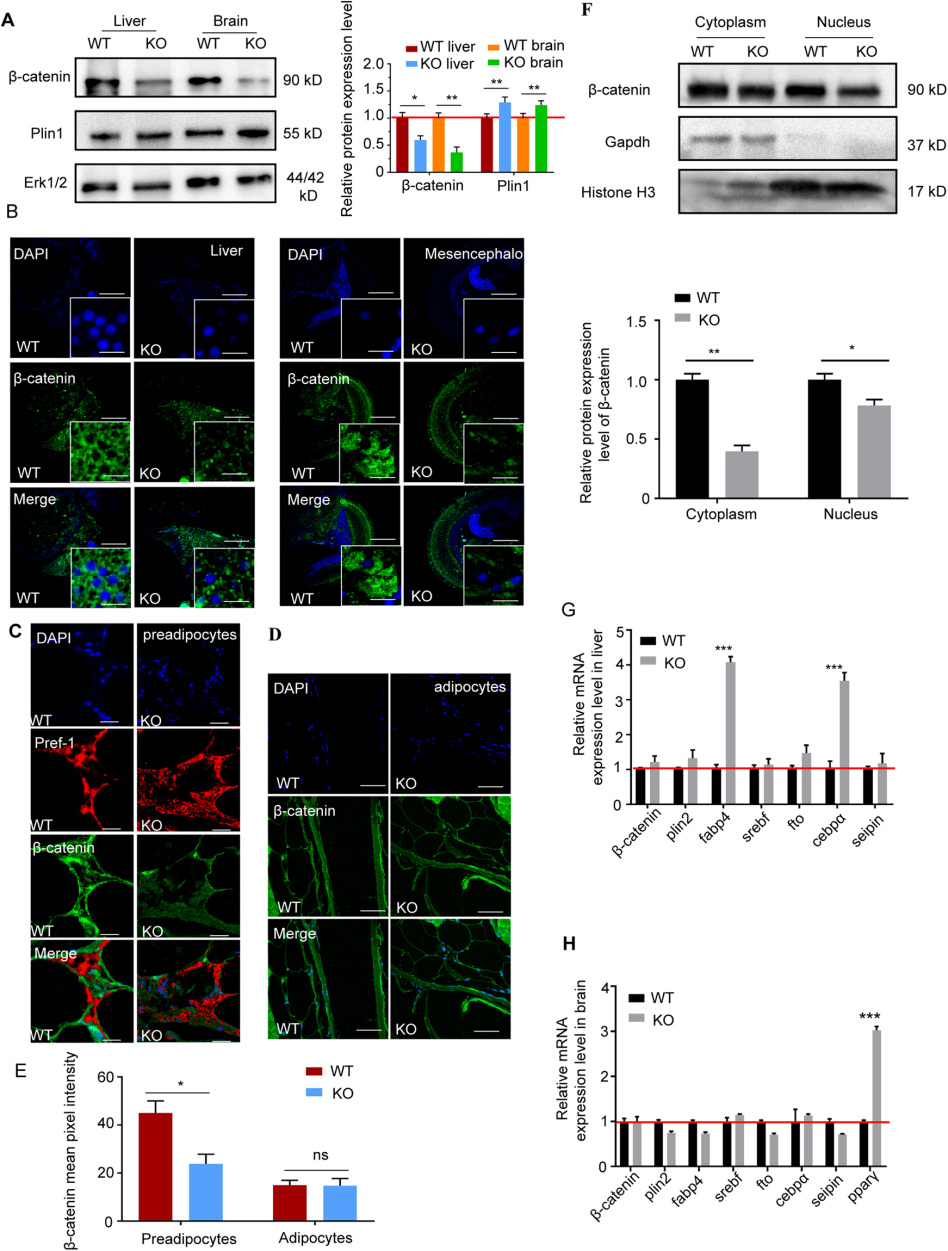
In zebrafish, *osbpl2b* deficiency reduces the protein level of β-catenin and affects β-catenin target gene expression. **A** Lysates of the brain and liver in the WT group or KO group were immunoblotted for β-catenin, Plin1, and Erk1/2 detection (*n* = 3). The data were normalized with Erk1/2 control. **B** Tissue sections of mesencephalon and liver in the WT group or KO group were immunostained with anti-β-catenin (green). Scale bar, 50 μm (inserts, 10 μm). **C** Tissue sections of adipocytes in the WT group or KO group were immunostained with anti-β-catenin (green), Pref-1 (red), and DAPI (blue). Pref-1 was used as a preadipocyte marker. Scale bar, 50 μm. **D** Tissue sections of preadipocytes in the WT group or KO group were immunostained with anti-β-catenin (green). Scale bar, 50 μm. **E** Mean fluorescence intensity was measured to show the expression level of β-catenin in the WT cells or KO cells shown in **C**, **D**. **F** Brains of the WT group and KO group were lysed, and the lysates were separated into cytosol and nucleus fractions. The abundance of β-catenin in the two fractions was assessed by western blotting. Histone H3 acted as internal nucleus control, and Gapdh acted as internal cytoplasm control (*n* = 3). **G**, **H** Real-time RT-PCR was used to analyze the mRNA levels of adipocyte differentiation-related genes in the brain and liver of the WT group or KO group (*n* = 3). Identification of Osbpl2b in the WT and KO zebrafish was showed in Supplementary Fig. S4. All data are from three independent experiments. The data are presented as the mean ± SD values (*n* ≥ 3). *P < 0.05; **P < 0.01, ***P < 0.001, ns: not significant.

We found that β-catenin decreased in liver and brain of the 4 mpf *osbpl2b*^−^^/−^ (KO) group compared with that in the WT group, indicating that the deletion of *osbpl2b* caused a reduction in β-catenin in multiple organs (Fig. 5A, B and Supplementary Fig. S4c). Furthermore, the immunofluorescence results showed that *osbpl2b* deficiency reduced β-catenin reduced in preadipocytes, but β-catenin was present at low levels in mature adipocytes in the WT and KO groups (Fig. 5C–E). Consistent with the in vitro experiments, we found that Osbpl2b co-localized with β-catenin in the liver (Supplementary Fig. S4d, e), and β-catenin was reduced in the nucleus of the KO group compared with that in the WT group (Fig. 5F). As shown in Fig. 5G, the deletion of *osbpl2b* in the liver upregulated multiple adipogenic-related genes, such as *fabp4* and *cebpα*, while the mRNA expression level of *β-catenin* showed no significant changes. In addition, the qRT-PCR results showed that only *ppar-γ* increased in the brain (Fig. 5H).

Taken together, these data confirmed that Osbpl2b co-localized with β-catenin and that *osbpl2b* deficiency reduced β-catenin in different tissues and affected the expression of downstream genes.

### Increased size and number of adipocytes in larvae and adult fish in the KO group

**Fig. 6:**
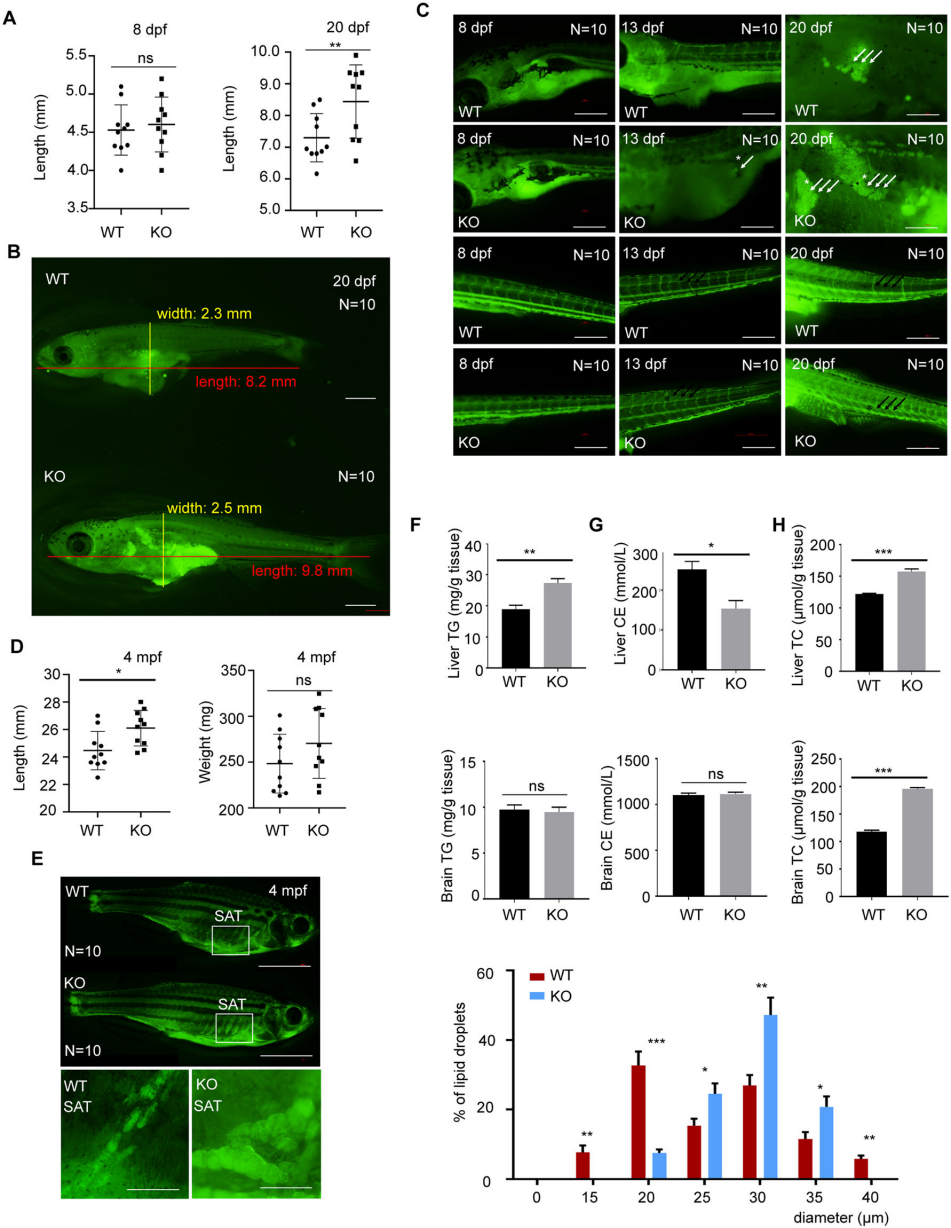
Deletion of *osbpl2b* increases both the size and number of adipocytes in larvae and adult zebrafish. **A** Larvae 8 dpf and 20 dpf were measured to determine their body lengths (*n* = 10). **B** Larvae 20 dpf were stained with BODIPY 493/503 to show body length and adipocyte distribution. Scale bar: 1000 μm. **C** Larvae at the indicated periods were stained with BODIPY 493/503 to show adipocyte LD size and number. White arrows: adipocyte, black arrows: blood vessel. Scale bar: 100 μm. **D** Adult zebrafish 4 mpf were measured to show body length and weight (*n* = 10). **E** Adult zebrafish 4 mpf were stained with BODIPY 493/503 to determine adipocyte LD size and number. Scale bar: 5 mm (inserts, 100 μm). **F** The TG concentrations of liver and brain in the 4 mpf WT group and KO group were detected by TG biochemical detection assay (*n* = 3). **G** The CE concentrations of liver and brain in the 4 mpf WT group and KO group were detected by CE ELISA assay (*n* = 3). **H** The TC concentrations of liver and brain in the 4 mpf WT group and KO group were detected by TC biochemical detection assay (*n* = 3). All data are from three independent experiments. The data are presented as the mean ± SD values (*n* ≥ 3). *P < 0.05; **P < 0.01, ***P < 0.001, ns: not significant.

The in vivo results showed a similar trend to that our previous in vitro experiments. We next focused on whether this molecular mechanism could induce preadipocyte differentiation during the development of zebrafish. It has been reported that the development of adipocytes in larvae is associated with body size^38,39^. Therefore, we predicted that a phenotype of both increased body length and adipocyte expansion would be observed in the zebrafish. We found that the KO larvae were much longer than the WT larvae 20 dpf (Fig. 6A, B), and the KO larvae initially formed adipocyte LDs much earlier than the WT larvae: adipocyte LDs in the KO larvae were observed 13 dpf, but not in the WT larvae. More adipocyte LDs were also found in the KO larvae than in the WT group 20 dpf during the developmental progression of the larvae (Fig. 6C). We also observed fluorescence in the blood vessels of the KO larvae, suggesting that the deletion of *osbpl2b* caused lipid deposition (Fig. 6C). All these results indicated that *osbpl2b* deficiency caused an increase in the number of adipocyte LDs in the larvae.

To verify whether *osbpl2b* deletion leads to obesity-related characteristics in adult zebrafish, the body length and weight of 4 mpf zebrafish were measured, and fluorescence staining was also performed. Consistent with the results of the larvae, the bodies in the adult KO group were longer than those in the adult WT group, although no significant difference in weight was found (Fig. 6D). Fluorescence staining showed that more adipocyte LDs were observed in the subcutaneous adipose tissue (SAT) of the KO group than in that of the WT group (Fig. 6E). The population of LDs with a diameter of 30 μm was increased in the KO cells, and the population of LDs with a diameter of 20 μm was decreased (Fig. 6E). Furthermore, the TG, cholesterol ester (CE), and total cholesterol (TC) levels in the liver and brain were tested to determine whether the enlarged LDs in the KO zebrafish were caused by an elevated level of TG and not by CE. As expected, we found that the TG and TC levels of the liver in the KO group were both increased compared with those in the WT group, while the CE level was decreased in the KO group (Fig. 6F–H). The TC level of the brain was increased in the KO group compared with that in the WT group. However, no significant difference in the TG and CE levels in the brain was found between the WT group and the KO group (Fig. 6F–H).

## Discussion

Our study indicates a novel function of OSBPL2/ORP2 in the developmental progression of preadipocytes: (i) OSBPL2/ORP2 binds to β-catenin and maintains β-catenin in the cytoplasm, (ii) the complex mediates β-catenin transfer into the nucleus and thus regulates the transcription and activation of adipogenic genes. (iii) Deletion of *OSBPL2* increases the ubiquitylation levels of β-catenin, leads to a decrease in β-catenin in the cytoplasm, reversing the suppression of adipogenic genes in the nucleus, (iv) and triggering preadipocytes to mature into adipocytes, finally leading to obesity-related characteristics and metabolic disorders. The above findings link OSBPL2/ORP2 to adipocyte differentiation and demonstrate that OSBPL2/ORP2 controls the progression of preadipocyte development, which provides a new molecular model for further understanding preadipocyte development and adipocyte differentiation. It has been reported that the ORP11 level increases in differentiated SGBS adipocytes, while ORP3 and ORP8 levels decrease^40^. The ORP11 level decreases in adipocyte tissue under caloric restriction, which may be due to fat mobilization^41^. However, few studies of OSBPL2/ORP2 in adipocytes or adipocyte tissue have been reported. In this study, we show that *Osbpl2* deficiency increases the number of LDs in 3T3-L1 preadipocytes when the cells are cultured for 8 d.

The interaction between OSBPL2/ORP2 and β-catenin also provides a clue for follow-up research on preadipocyte development. This finding supports the function of β-catenin in suppressing adipogenesis^42^, and the stabilization of β-catenin results in a failure of PPAR-γ and C/EBPα activation in preadipocytes^43,44^. A recent study showed that raloxifene could reduce fat accumulation and suppress the activation of adipogenic factors by preserving the expression of canonical Wnt10b/β-catenin^45^. Moreover, carbamazepine inhibits Wnt/β-catenin expression and enhances adipogenesis^46^. In addition to the function of β-catenin in adipogenesis, a disruption of OCT4 and β-catenin recruitment to target gene promoters may affect differentiation, pluripotency, and cell cycle genes^47^. A previous study showed that elevating the ORP8 level could significantly reduce the expression levels of Wnt3a and β-catenin^48^. Our findings show that, in contrast to ORP8, OSBPL2/ORP2 interacts with β-catenin, and a lack of OSBPL2/ORP2 leads to a decrease in β-catenin.

In this study, we found that OSBPL2/ORP2 maintained β-catenin in the cytoplasm and promoted β-catenin translocation into the nucleus, indicating the underlying molecular mechanism by which OSBPL2/ORP2 and preadipocyte development are related to obesity. It has been reported that USP20 controls the stability of β-catenin and prevents it from degrading by deubiquitinating β-catenin^35^. In addition, ORP8 is reported to interact with USP5, and this interaction causes ORP8 deubiquitination and accumulation and leads to growth inhibition^49^. Interestingly, by mass spectrometric analysis, we also identified several members of the USP family (USP10, USP14, USP16, and USP39) among the binding partners of OSBPL2/ORP2, suggesting an underlying relationship among OSBPL2/ORP2, β-catenin, and member of the USP family.

In addition, we demonstrate that OSBPL2/ORP2 contributes to the developmental progression of preadipocytes using the 3T3-L1 cell line and a zebrafish model. In the previous study, we described the association of *OSBPL2* mutations with autosomal dominant nonsyndromic hearing loss in a large affected Chinese family^50^, which were then verified in other affected families.^51,52^. To confirm the effect of *OSBPL2* disruption on hearing loss, we established *OSBPL2*-disrupted Bama mini pig models. In addition to the expected phenotype of hearing loss, we found the obesity phenotypes with hypercholesterolemia in the *OSBPL2* mutation Bama mini pig^16^. Using HepG2 cells and zebrafish, we have reported that deletion of *OSBPL2* affected LD lipolysis^15^. All these studies suggest that OSBPL2 is a gene/protein with multiple functions. We showed that the absence of OSBPL2/ORP2 caused a decrease in β-catenin, but the mechanism by which β-catenin is reduced was still needed to be further investigated in future work.

Overall, this work revealed an underlying molecular mechanism by which OSBPL2/ORP2 regulates preadipocytes development by maintaining β-catenin in the cytoplasm and participating in the translocation of β-catenin into the nucleus. Our work presented a novel insight into the function of OSBPL2/ORP2, which may provide the potential target and therapeutic approach in the treatment of obesity-related metabolic disease.

## Supplementary information

Supplementary Figure Legends

Figure S1

Figure S2

Figure S3

Figure S4

Table S1

## Data Availability

All data generated or analyzed during this study are included in this published article.
