# Supplementary material for: Oxysterol-binding protein-like 2 contributes to the developmental progression of preadipocytes by binding to β-catenin
Source: Cell Death Discov. 2021 May 17;7:109. doi: 10.1038/s41420-021-00503-2 (PMC8129138; doi:10.1038/s41420-021-00503-2)
Supplement: Supplementary file 1 — Supplementary Figure Legends [file 41420_2021_503_MOESM1_ESM.docx]

**Supplementary Figure Legends**

**Fig. S1 The establishment of stable *Osbpl2*-deficient 3T3-L1 cell lines with CRISPR-Cas9 gene editing technique.** Related to ***Fig. 1****.* **a** Scheme diagram representing the amino acid sequence of OSBPL2 in the KO cells and the WT cells. **b** Identification of OSBPL2 at the protein expression level was performed by western blotting. **c** Real-time RT-PCR was used to analyze the mRNA levels of adipocyte differentiation-related genes in the 3T3-L1 preadipocytes treated with MDI (n=3). MDI: 3-isobutyl-1-methylxanthine, dexamethasone, and insulin. **d, e** Lysates of the 3T3-L1 preadipocytes treated with MDI were immunoblotted for β-catenin, PPAR-γ, PLIN1, OSBPL2 and ERK1/2 detection (n=3).

**Fig. S2 OSBPL2/ORP2 is associated with the developmental progression of preadipocytes presented with morphology**. Related to ***Fig. 1****.* **a-d** WT cells and KO cells were treated with different inducers for 48 h and then fixed and stained with BODIPY 493/503 (green) and DAPI (blue) to determine the dynamic changes of LDs. Scale bar, 50 μm. **c** WT cells and KO cells were treated with MDI for 48 h, and observed in light bright screen. Scale bar, 50 μm. **d** WT and KO cells were treated with different inducers for 48 h and cultured for the indicated periods. The cells were then fixed and stained with oil red-O solution to determine the LDs. MDI: 3-isobutyl-1-methylxanthine (M), dexamethasone (D), and insulin (I). Scale bar, 50 μm.

**Fig. S3 OSBPL2/ORP2 binds to β-catenin and regulated the ubiquitination level of β-catenin**. Related to ***Fig. 2-4****.* **a** Co-IP assays were used to verify the interaction of endogenous Osbpl2 with endogenous β-catenin in 3T3-L1 preadipocytes. **b** WT cells and KO cells were induced by MDI for 48 h and then cultured 8 d. The cells were fixed and immunostained with anti-β-catenin (red). Scale bar, 50 μm (inserts, 10 μm). Mean fluorescence intensity was measured to show the expression level of endogenous β-catenin in the WT cells and KO cells. **c** 3T3-L1 preadipocytes were treated with 20 μM SKL2001 for 24 h The concentration of cholesterol in the WT cells and KO cells was detected with a cholesterol biochemical detection assay (n=3). **d, e** WT and KO cells were treated with or without 20 μM SKL2001 and cultured for 8 d along with DI treatment. The cells were then fixed and stained with oil red-O solution or BODIPY 493/503 dye to determine the LDs. Scale bar, 50 μm. All data are from three independent experiments. The data are presented as the mean ± SD values (n ≥ 3). **P* ＜ 0.05; ***P* ＜ 0.01, ****P* ＜ 0.001, ns: not significant.

**Fig. S4 In zebrafish, Osbpl2b co-localizes with β-catenin.** Related to ***Fig. 6****.* **a** Sequencing chromatograms of *osbpl2b* allelic mutations in the KO zebrafish compared with the WT zebrafish. **b** Identification of Osbpl2b at the protein expression level was performed by western blotting. **c** Tissue sections of the mesencephalon and liver in the WT group or KO group were immunostained with anti-β-catenin using immunohistochemistry. Scale bar, 50 μm. **d** Tissue sections of the mesencephalon and medulla oblongata in the WT zebrafish were immunostained with anti-β-catenin or anti-Osbpl2b using immunohistochemistry. Arrows pointed the regions of the mesencephalon and medulla oblongata that β-catenin or Osbpl2b localized. Scale bar, 50 μm. **e** Tissue sections of the mesencephalon and liver in the WT zebrafish were immunostained with anti-β-catenin (green) and anti-Osbpl2b (red). Scale bar, 50 μm (inserts, 10 μm).
