# Supplementary figures and images for: Oxysterol-binding protein-like 2 contributes to the developmental progression of preadipocytes by binding to β-catenin
Source: Cell Death Discov. 2021 May 17;7:109. doi: 10.1038/s41420-021-00503-2 (PMC8129138; doi:10.1038/s41420-021-00503-2)

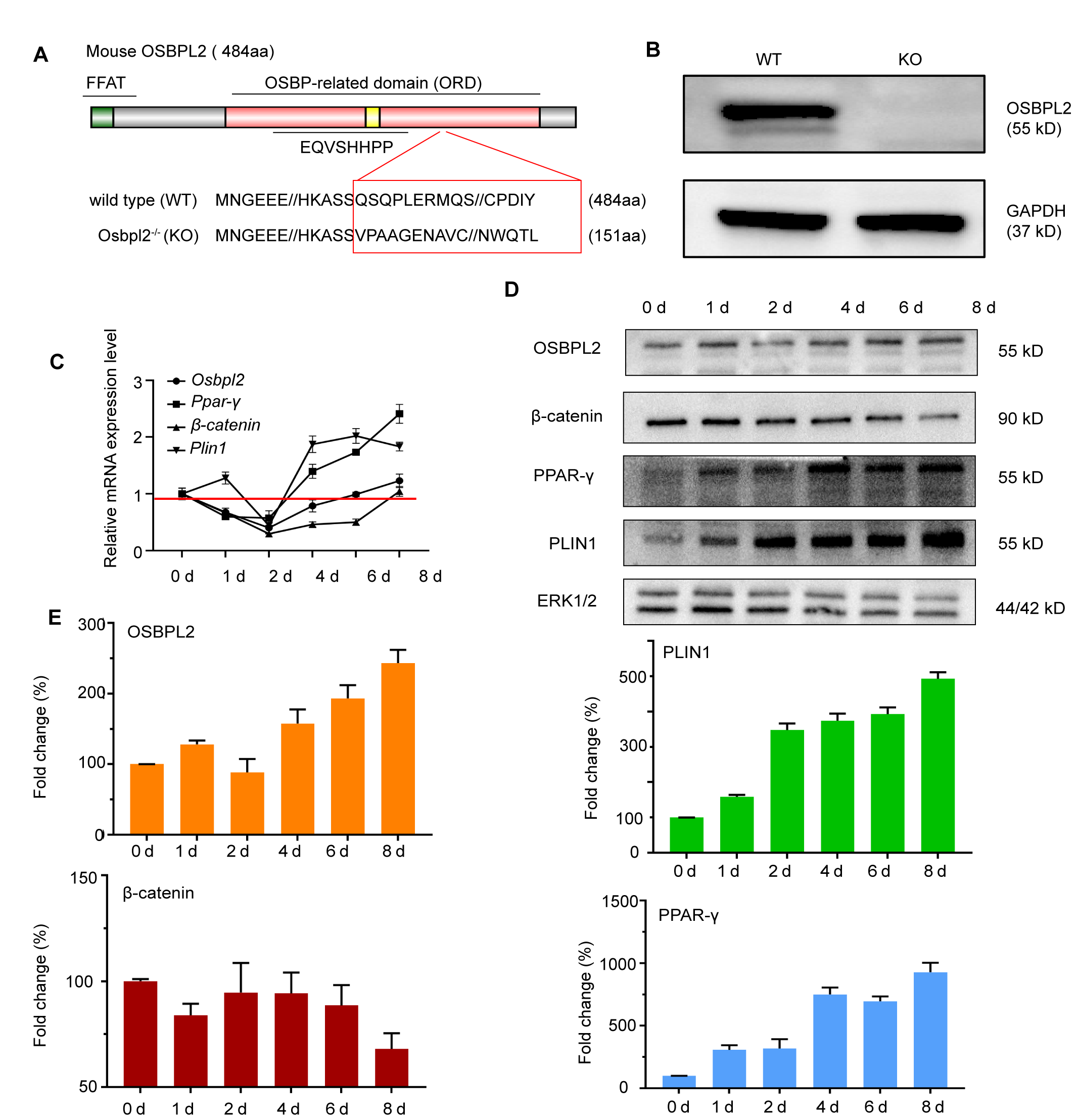

Supplement: Supplementary file 2 — Figure S1 [file 41420_2021_503_MOESM2_ESM.tif]

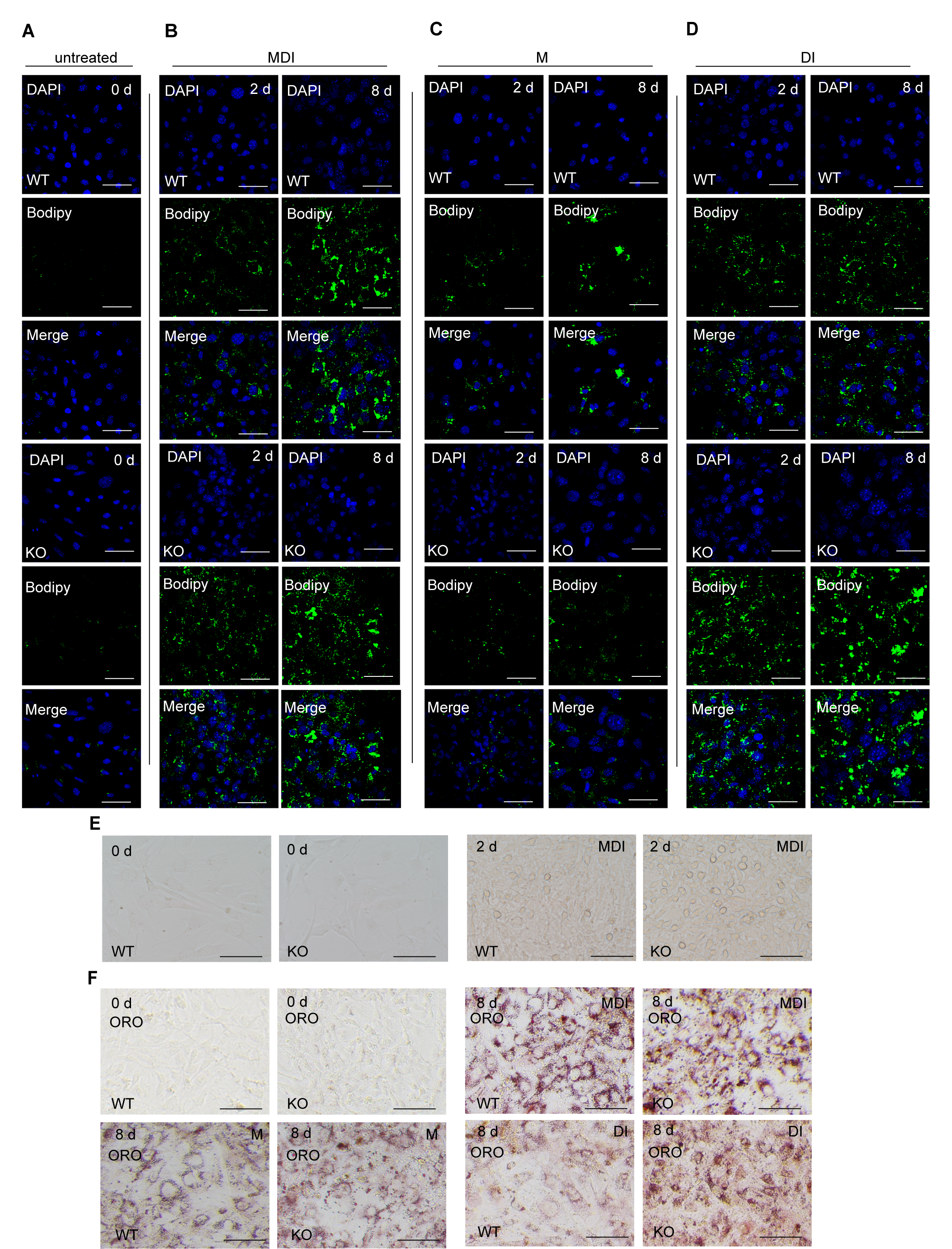

Supplement: Supplementary file 3 — Figure S2 [file 41420_2021_503_MOESM3_ESM.tif]

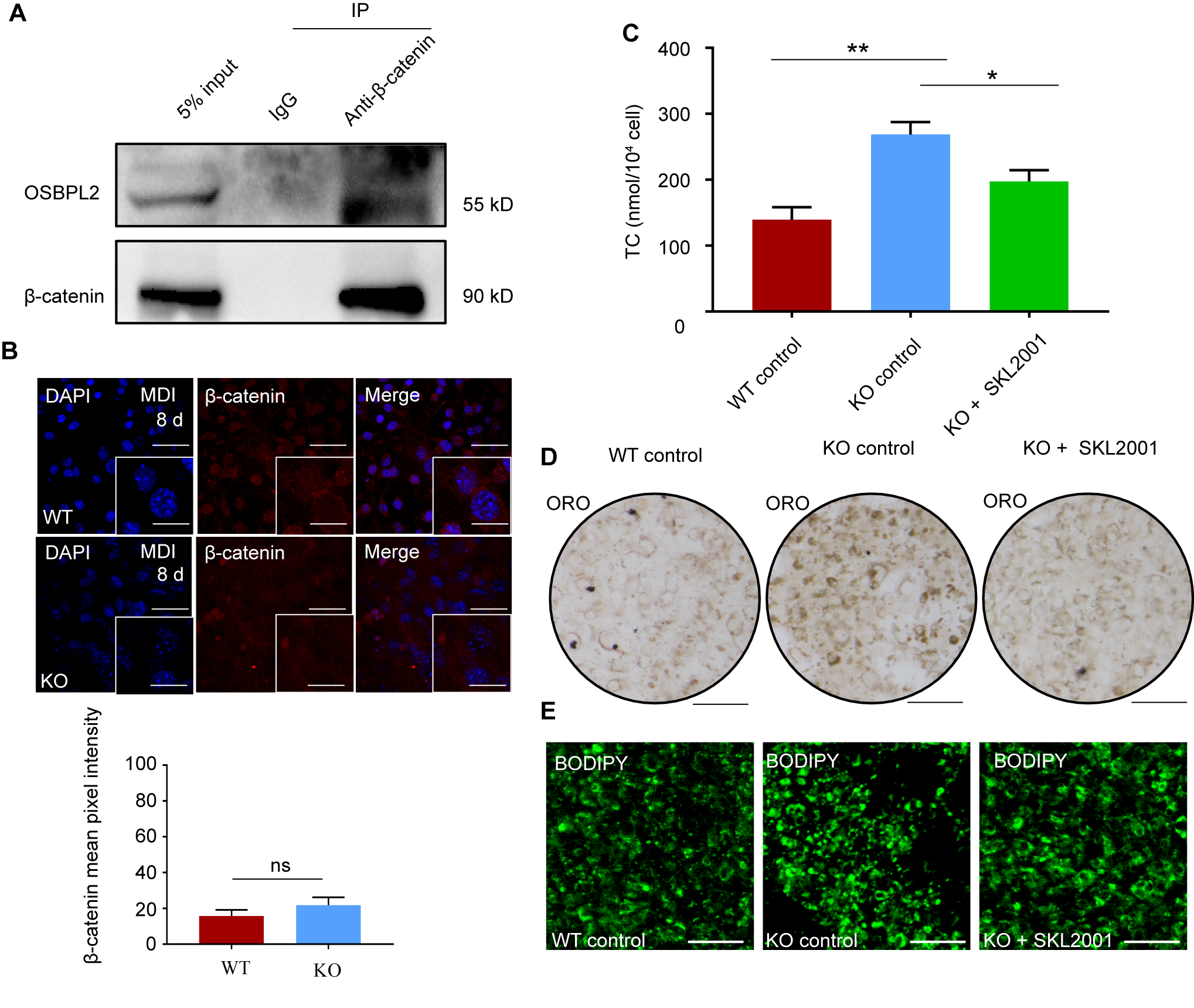

Supplement: Supplementary file 4 — Figure S3 [file 41420_2021_503_MOESM4_ESM.tif]

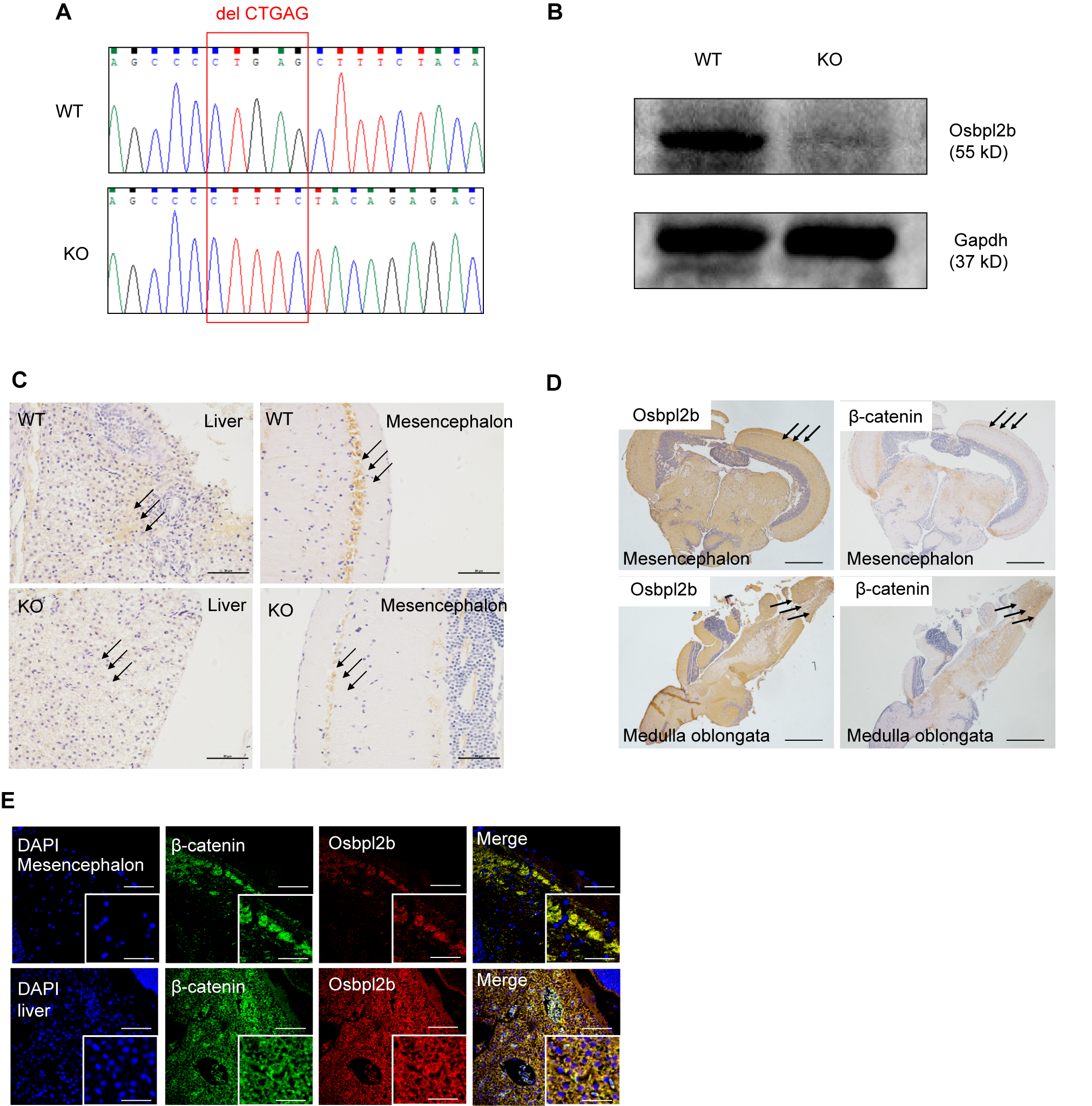

Supplement: Supplementary file 5 — Figure S4 [file 41420_2021_503_MOESM5_ESM.tif]
