# Supplementary material for: Oxysterol-binding protein-like 2 contributes to the developmental progression of preadipocytes by binding to β-catenin
Source: Cell Death Discov. 2021 May 17;7:109. doi: 10.1038/s41420-021-00503-2 (PMC8129138; doi:10.1038/s41420-021-00503-2)
Supplement: Supplementary file 6 — Table S1 [file 41420_2021_503_MOESM6_ESM.docx]

**Table S1 Primer sequences.**

| Gene |  | primer |
| --- | --- | --- |
| Plin1 (mouse) | Forward | 5′-CGTGGAGAGTAAGGATGTCAATG-3′ |
|  | Reverse | 5′-GGTGCTGTTGTAGGTCTTCTG-3′ |
| Plin2 (mouse) | Forward | 5′-GTTAGGCGTCTCTTTTCTCCAG-3′ |
|  | Reverse | 5′-TCCTTTGTACTGACATAAGCGG-3′ |
| FABP4 (mouse) | Forward | 5′-GACAGGAAGGTGAAGAGCATC-3′ |
|  | Reverse | 5′-CACGCCTTTCATAACACATTCC-3′ |
| Pparγ (mouse) | Forward | 5′-TGTTATGGGTGAAACTCTGGG-3′ |
|  | Reverse | 5′-AGAGCTGATTCCGAAGTTGG-3′ |
| Srebf1 (mouse) | Forward | 5′-GCTTAGCCTCTACACCAACTG-3′ |
|  | Reverse | 5′-GTGAGCTACCTGGACTGAAG-3′ |
| Fto (mouse) | Forward | 5′-CCTCAATGACTCAGACGATGG-3′ |
|  | Reverse | 5′-CGATTGCCTTGAAACCAGAAC-3′ |
| Cebpα (mouse) | Forward | 5′-AGAGCCGAGATAAAGCCAAAC-3′ |
|  | Reverse | 5′-TCATTGTCACTGGTCAACTCC-3′ |
| Seipin (mouse) | Forward | 5′-AACTTCACGTTCCTCAGCG-3′ |
|  | Reverse | 5′-TGTGAGTTATCCCTTTGTCGG-3′ |
| Srebf1 (mouse) | Forward | 5′-GCTTAGCCTCTACACCAACTG-3′ |
|  | Reverse | 5′-GTGAGCTACCTGGACTGAAG-3′ |
| Osbpl2 (mouse) | Forward | 5′-CTTGTGGATTGTTTGGGAAGG-3′ |
|  | Reverse | 5′-GGACTCGTATGAAGCAGGATC-3′ |
| β-catenin (mouse) | Forward | 5′-GCTATTCCACGACTAGTTCAGC-3′ |
|  | Reverse | 5′-AGCTCCAGTACACCCTTCTAC-3′ |
| plin1 (zebrafish) | Forward | 5′-AAACCTCTCGAACCAGCTTC-3′ |
|  | Reverse | 5′-CTTCTGTCTTGGATAGTCGCC-3′ |
| plin2 (zebrafish) | Forward | 5′-AGGTTGCTGAGAATGGTGTC-3′ |
|  | Reverse | 5′-GTAGGATGGGCAGAGTCTTTTC-3′ |
| fabp4 (zebrafish) | Forward | 5′-CGAGTACATGAAGGCTATAGGTG-3′ |
|  | Reverse | 5′-GTGGTTTTGAAGGTGCTCTG-3′ |
| pparγ (zebrafish) | Forward | 5′-GGAGAACACATACAGAGCACAG-3′ |
|  | Reverse | 5′-CAGTGAGGATGAAGACGGATC-3′ |
| fto (zebrafish) | Forward | 5′-CTCAAGACTACATAACCCAGCG-3′ |
|  | Reverse | 5′-TCTGCTCTCCTAGTTCCTGTAG-3′ |
| cebpα (zebrafish) | Forward | 5′-CAACCCAAACACCAACACTG-3′ |
|  | Reverse | 5′-TCTTTACTCACACCTCGCTTC-3′ |
| seipin (zebrafish) | Forward | 5′-GATTCAGTCTCGCAGGGTTC-3′ |
|  | Reverse | 5′-GCACAATCACACTGAGGAATG-3′ |
| srebf1 (zebrafish) | Forward | 5′-AAAGCCATCGAGTACATCCG-3′ |
|  | Reverse | 5′-CACCAGATCCTTCAGAGACTTG-3′ |
| β-catenin (zebrafish) | Forward | 5′-ACCCTACACAATCTTTCCCAC-3′ |
|  | Reverse | 5′-AAACAGCACAGAGTCCACAG-3′ |
| kctd15 (zebrafish) | Forward | 5′-TCACTAAATCCAACGCTCCAG-3′ |
|  | Reverse | 5′-CTGTCCAAAACAATCGGTTCG-3′ |
| gnpda2 (zebrafish) | Forward | 5′-CTGACCGCTACTTCACTTTAGG-3′ |
|  | Reverse | 5′-CAGACCACATACTCGTCCATG-3′ |
| Sh2b1 (zebrafish) | Forward | 5′-CGGTTCAGATGAGAGCGTG-3′ |
|  | Reverse | 5′-ACCATAGCACACTTGAGACAG-3′ |
| mtch2 (zebrafish) | Forward | 5′-ATTAACAGTCCTTTCCCACCC-3′ |
|  | Reverse | 5′-AAAGCCCTGGAAGTTGGTAG-3′ |
| negr1(zebrafish) | Forward | 5′-AGTTTTCGAGTGGTACAAGGG-3′ |
|  | Reverse | 5′-CGATCTGGAGCTGAGGTTATTG-3′ |
